# Autoimmune hemolytic anemia associated with babesiosis

**DOI:** 10.1186/s40364-017-0095-6

**Published:** 2017-04-08

**Authors:** Roshni Narurkar, Aleksandra Mamorska-Dyga, John C. Nelson, Delong Liu

**Affiliations:** Department of Medicine, New York Medical College and Westchester Medical Center, Valhalla, NY 10595 USA

## Abstract

**Background:**

Babesiosis is endemic in selected areas in North America. Babesia infection is commonly associated with anemia, thrombocytopenia, hyponatremia and elevated liver enzymes. Autoimmune hemolytic anemia (AIHA) is known to be caused by parasitic and viral infections but has not been well characterized.

**Case presentation:**

We describe two cases diagnosed with babesiosis triggering severe AIHA. One case had history of splenectomy, and the other was an elderly patient. Older, immunocompromised and asplenic patients may be particularly at risk for post-babesiosis AIHA (PB-AIHA).

**Conclusions:**

The pathogenesis for conventional AIHA and PB-AIHA appears to be different, since splenectomy is a treatment for conventional AIHA, whereas PB-AIHA is seen more often in asplenic patients. Further investigation into this intriguing mechanism of host immune response to babesiosis may help to elucidate the overall mechanism of infection- triggered AIHA.

## Background

Human babesiosis was first reported in 1957 [1, 2]. Babesia divergens is common in Europe, while babesia microti is more commonly seen in North America [3]. In the Unites States 97% of the reported cases came from seven North-East states, occurring predominantly in the months of June–August [4, 5]. According to the CDC data, 1744 cases of babesiosis were identified in 2014, with almost all of them diagnosed in the Northeastern United States and New England. Babesia microti is spread in nature by *ixodes scapularis* ticks (also called blacklegged ticks or deer ticks). Babesia infection may vary from an asymptomatic course to a life-threatening disease [4, 6, 7]. Immunocompromised and asplenic patients are especially susceptible to serious illnesses. Common laboratory findings include hemolytic anemia, thrombocytopenia, hyponatremia and elevated liver enzymes [8–12].

Autoimmune hemolytic anemia (AIHA) is characterized by shortened red blood cell (RBC) survival and presence of auto-antibodies with a positive direct antiglobulin test (DAT) [13, 14]. DAT detects immunoglobulin and/or complement C3d against autologous RBCs [15]. The binding pattern is suggestive of specific etiologies. For example, IgG alone or IgG and C3d binding is seen in warm AIHA and drug induced anemias, as opposed to C3d alone, which is seen in cold agglutinin disease. AIHA can be idiopathic or secondary to lymphoproliferative disorders, malignancies, autoimmune disorders and infections. Lymphoproliferative disorders are the most common causes of both types of AIHA. Autoimmune diseases are associated with warm AIHA, while infections cause cold AIHA [16]. AIHA is known to be caused by parasitic and viral infections. Here, we report 2 cases of babesiosis complicated with severe warm autoimmune hemolytic anemia.

## Case presentation

### Case 1

A 43 year old male had history of hemophagocytic lymphohistiocytosis (HLH) 9 years ago who had splenectomy at that time. The patient was admitted to an outside hospital with complaints of persistent fever, malaise, nausea and vomiting. He was diagnosed with babesiosis and ehrlichiosis [17, 18]. He was treated with antibiotics including IV azithromycin, doxycycline and atovaquone for babesiosis and ehrlichiosis [18–21]. He was discharged home on oral antibiotics [22].

Shortly after the discharge, he presented with dark urine to the local hospital with laboratory findings suggestive of hemolytic anemia. He was then transferred to our institution. Upon admission laboratory values were significant for total bilirubin of 5.4 mg/dL, (direct bilirubin of 0.6 mg/dL), white blood cell count (WBC) of 16.9 × 10^3^/mm^3^ (30% neutrophils, 53% lymphocytes, and 15% monocytes), reticulocyte count of 13%, lactate dehydrogenase (LDH) level of 884 U/L and haptoglobin measured as <8 mg/dL. Ferritin level was 4527 μg/L, triglyceride 265 mg/dL. We performed bone marrow aspiration and biopsy, which revealed trilineage hematopoiesis with erythroid hyperplasia, as well as presence of Babesia species in mature erythrocytes (1–2%). Immunohistochemistry stains demonstrated CD163 positive macrophages engulfing erythrocytes and neutrophils, consistent with hemophagocytosis [23, 24]. Patient was started on chemotherapy for HLH with etoposide and steroids with overall improvement in laboratory values and clinical condition. He also continued to receive antibiotics for treatment of babesiosis with reduction and eventual clearance of blood parasitemia. The hospital course was complicated by deep vein thrombosis in lower extremity, for which patient was treated with heparin and warfarin. One week post discharge, he was again admitted to our hospital with complaints of vomiting, nausea and discoloration of urine. Laboratory data on second admission was significant for total bilirubin of 6.3 mg/dL (direct bilirubin of 0.6 mg/dL), LDH of 408 U/L and haptoglobin <8 mg/dL. Ferritin level was 4205 μg/L, triglyceride 373 mg/dL. Pathology from bone marrow aspiration and biopsy at this admission revealed hypocellular bone marrow with trilineage hematopoiesis, marginally increased monocytes/histiocytes and rare hemophagocytosis. Direct antiglobulin test (DAT) was positive (IgG and polyclonal, negative previously). Testing for paroxysmal nocturnal hemoglobinuria, cryoglobulinemia, glucose-6-phosphate dehydrogenase (G6PD) deficiency and hyperhomocystinemia was negative. Patient was maintained on methylprednisolone for AIHA. Blood parasite smear was negative for blood parasites, but babesia microti DNA PCR was persistently positive [25, 26], for which patient was continued on antibiotics. His overall status improved and patient was discharged home with oral antibiotics and prednisone. Prednisone was tapered to off over the following 6 weeks. The patient has not had recurrence of hemolysis for 14 months.

### Case 2

An 81 year old female with multiple medical conditions including non-obstructive coronary artery disease, hypertension and chronic bronchitis presented to an outside hospital with complaints of progressive fatigue, shortness of breath, associated with chills and diaphoresis. Immediately prior to admission she noticed yellow discoloration of the skin. The patient recalled history of a tick bite 1 month earlier, which was removed without residual skin rash. In the outside institution she was diagnosed with babesiosis, as well as acute kidney injury, liver insufficiency and non-ST elevation myocardial infarction. She was started on antibiotics and transferred to our hospital. Upon admission laboratory studies were significant for WBC of 7 × 10^3^/mm^3^, hemoglobin of 9.1 g/dL, platelet count of 54 × 10^3^/mm^3^, creatinine of 3.56 mg/dL, LDH 2161 U/L, aspartate transaminase (AST) of 271 U/L and alanine transaminase (ALT) of 143 U/L. Total bilirubin was 3.6 mg/dL (with direct bilirubin of 1.8 mg/dL) and haptoglobin was <8 mg/dL. Peripheral blood smear was positive for babesiosis with parasitemia of <1%. In the following days a decline in hemoglobin was noted to the lowest value of 6.5 g/dL. DAT was positive (IgG and polyclonal). The anemia was therefore consistent with AIHA. Patient’s renal function and respiratory status further deteriorated. She was intubated for acute respiratory distress syndrome and started on hemodialysis. She also received vasopressor support for a brief period of time. Patient underwent one session of plasmapheresis for severe AIHA. Blood parasitemia cleared but hemoglobin levels remained low despite transfusion of multiple units of cross-matched packed red blood cells. The patient’s status continued to deteriorate with progressive multiorgan failure leading to the decision of withdrawal of care. The patient subsequently expired.

## Discussion

This study reported two cases of severe AIHA associated with babesiosis. The babesiosis- associated AIHA remains rare, even though anemia is commonly seen in babesia infections [11, 27]. Although unlikely, it remains unclear whether erlichiosis had any role in triggering AIHA in the first case. AIHA has been reported in a variety of parasite infections. AIHA has been reported to be associated with acute malaria infection [28, 29]. Leishmania species causing Kala-azar disease is another intracellular parasite associated with DAT positive hemolytic anemia [30]. Viral infections including influenza virus, hepatitis virus, cytomegalovirus, Epstein-Barr virus have been implicated in AIHA [31]. Farwell et al. described Coombs positive anemia in dogs infected with Babesia gibsoni and Babesia canis [32]. Post transfusion babesiosis with a DAT positive hemolytic anemia has also been reported [33, 34]. It is important to consider blood parasite infections as a probable cause of hemoglobinuria and warm AIHA [15, 16]. AIHA was recently reported in 7% of all patients with babesia infection in a series, but close to 30% in asplenic patients [35]. The mechanism for post-babesiosis AIHA (PB-AIHA) was proposed to be related to antibody-mediated and immune-complex- mediated hypersensitivity. However, there is clear difference in the pathogenesis for conventional AIHA and PB-AIHA, since splenectomy is a treatment for conventional AIHA, whereas PB-AIHA is seen more often in asplenic patients. Further investigation into this intriguing mechanism of host immune response to babesiosis may help to elucidate the overall mechanism of infection- triggered AIHA.

## Conclusions

Severe AIHA may be triggered by babesiosis. Older and asplenic patients may be particularly at risk for post-babesiosis AIHA.

## Abbreviations

AIHA: Autoimmune hemolytic anemia
DAT: Direct antiglobulin test
HLH: Hemophagocytic lymphohistiocytosis
